# Relative Body Mass Index Improves the BMI Percentile Performance for Detection and Monitoring of Excess Adiposity in Adolescents

**DOI:** 10.3390/nu16050703

**Published:** 2024-02-29

**Authors:** Pedro A. Velasquez-Mieyer, Ramfis Nieto-Martinez, Claudia P. Neira, Diana De Oliveira-Gomes, Andres E. Velasquez Rodriguez, Eunice Ugel, Patricia A. Cowan

**Affiliations:** 1 Lifedoc Health, 6625 Lenox Park Drive, Suite 205, Memphis, TN 38115, USA; nietoramfis@hsph.harvard.edu (R.N.-M.); cneira@lifedochealth.org (C.P.N.); 2 Lifedoc Research, 6625 Lenox Park Drive, Suite 205, Memphis, TN 38115, USA; 3 Departments of Global Health and Population and Epidemiology, Harvard T. H. Chan School of Public Health, Harvard University, Boston, MA 02115, USA; eugelgarrido@hsph.harvard.edu; 4 Department of Internal Medicine, University of Texas Southwestern Medical Center, Dallas, TX 75390, USA; diana.deoliveiragomes@utsouthwestern.edu; 5 College of Nursing, University of Arkansas for Medical Science, Little Rock, AR 72205, USA; pacowan@uams.edu

**Keywords:** pediatric, obesity, adiposity, relative body mass index, body mass index, youth, dual energy X-ray absorptiometry (DXA)

## Abstract

Obesity is defined as excess adipose tissue; however, commonly used methods may under-detect adiposity in adolescents. This study compared the performance of body mass index percentile (BMI%) and relative body mass index (RBMI) in identifying excess body fat percentage (BF%) and estimated RBMI cut points to better stratify severity of adiposity. In 567 adolescents ages 11–19 year, BF% measured by DXA was used to compare BMI% and RBMI performance at different degrees of adiposity. RBMI cut points for adiposity detection were derived via ROC curve analysis. BF% was strongly correlated with BMI% (r = 0.889, *p* < 0.001) and RBMI (r = 0.901, *p* < 0.001). However, RBMI exhibited less dispersion and better discriminated the relationship with BF% independent of age, race, and gender. Both BMI% and RBMI performed similarly for detecting high BF% (≥25 BF% in males; ≥30 BF% in females). Nonetheless, the relationship of BMI% with BF% was diminished among leaner adolescents. RBMI detected overweight in 21.3% more females and 14.2% more males. RBMI improved the detection of excess adiposity in individuals otherwise classified as having normal weight or overweight by BMI%. RBMI is a valuable and accessible tool for earlier detection, intervention, and effective follow-up of excess adiposity in youth at higher risk for complications.

## 1. Introduction

Children and adolescents are increasingly susceptible to obesity-related complications in early adulthood such as type 2 diabetes, hypertension, chronic kidney disease, coronary artery disease, sleep apnea, heart failure, musculoskeletal disorders, and several cancers. In the US, 14.7 M (19.7%) children and adolescents meet the diagnostic criteria of obesity, defined as a body mass index (BMI) >95th percentile for age and sex [1]. Childhood obesity tracks into adulthood, with obese children and adolescents being five times more likely to be obese in adulthood than youth who were not obese [2]. Using simulated growth trajectories models, a recent study predicted that >50% of today’s children will be obese at the age of 35, indicating that excess adiposity in childhood will continue to be a major health problem in the US [3]. Accurate and timely detection of obesity in children is needed to treat excess adiposity and prevent the myriad of obesity-related conditions.

In children and adolescents, BMI percentiles (BMI%) are commonly used to assess adiposity. However, as youth transition through puberty into young adulthood, the relationship between BMI% and adiposity is skewed, a phenomenon not fully addressed by BMI percentiles (BMI%) [4]. BMI%-based categories misclassify youth who do not have excess adiposity in terms of body fatness. BMI% does not always reflect underlying adiposity, and these findings reinforce the need to consider alternative measures to percentile categories when studying adiposity in children and adolescents [5,6,7].

While reference percentiles for BMI in children and adolescents are established, reference data for BF% in youth are limited. Abnormal BF% levels can be estimated using the population distribution or the identification of adverse biological endpoints. Various cut points of BF% estimated by skinfold thickness (≥20% and ≥25% in males, and ≥30% in females) to detect high blood pressure and dyslipidaemia in youth have been proposed [8,9]. A meta-analysis of 53,521 children between 4 and 18 years of age demonstrated that the most commonly used BF% cut points to define obesity by (dual energy X-ray absorptiometry) DXA were ≥25% in males, ≥30% in females, and ≥95th percentile BF% in youth of both sexes [10].

Cole and colleagues [11] have suggested that changes in growing children’s adiposity be evaluated using the percentage difference from the sex-age-specific median BMI. However, a comparison of median BMI to DXA measures of fat mass was not performed. The practicality of calculating the natural log of BMI/median BMI in a clinical setting is also limited. Thus, further investigation of clinically useful measures for assessing and tracking adiposity in children that reflect DXA measures of fat mass [BF%] is needed. Our team has proposed the use of relative body mass index (RBMI) as a clinical estimation of the percentage of overweight above the ideal (50th) BMI% for age and sex [12]. In a biracial sample of adolescents, we previously reported the use of arbitrarily selected RBMI cut points to uncover an overweight threshold for the deterioration of glucose metabolism (RBMI: ≥125 and <150%, ≥150 and <175%, ≥175 and <200%, ≥200 and <250%, and ≥250%). Adolescents with an RBMI >150% exhibited severe deterioration of insulin sensitivity (~55%) as assessed by the Composite Whole Body Insulin Sensitivity Index (CISI), the single best predictor of impaired glucose metabolism (as prediabetes and diabetes by oral glucose tolerance test) [13,14].

Thus, this study aims to elucidate discrepancies in the relationships between BMI% as well as RBMI and BF% measurement by DXA in a sample of adolescents. We also sought to identify specific RBMI cut points to more effectively define and stratify the severity of adiposity. We hypothesize that, compared to BMI%, RBMI will better categorize adolescents with excess adiposity, especially at lower levels. The use of RBMI will also facilitate the evaluation of adiposity at different growth stages and across race, age, and sex.

## 2. Materials and Methods

### 2.1. Study Design and Population

Data were pooled from four studies of healthy AA and Caucasian children and adolescents (n = 567) aged 11 to 19 who underwent a DXA scan and had anthropometric measurements. Youth who weighed ≥160 kg (upper weight limit for DXA equipment), who had casts or missing limbs, or who were pregnant were excluded in the original studies.

### 2.2. Study Variables

Race, sex, and date of birth were self-reported by youth and validated by parents/legal guardians. Female participants underwent urine pregnancy testing. Height was measured using a Digital Stationary Stadiometer (Seca^®^ 264, Chino, CA, USA) to the nearest 0.1 cm, while weight was measured using a calibrated Digital Platform Scale (Health-O-Meter^®^ Pro Plus 2101KL, McCook, IL, USA) to the nearest 0.1 kg. Whole body dual energy X-ray absorptiometry (DXA) scans were performed by a DXA-certified health care professional following a standardized protocol using the Hologic Discovery A software, v8.3 (Hologic^®^, Bedford, MA, USA). Quality control measures included daily equipment calibration with scanning of phantoms. Participants were positioned on the DXA table in a supine position with toes pointing together and secured lower legs to reduce movement. Participants who were too large to fit within the limits of the scan region were scanned with the correct placement of the right arm and part of the left arm out of the region of interest; then left-arm values were substituted for the right-arm values. The DXA scans provided soft tissue (fat and lean mass) and bone mineral measurements. DXA measures of adiposity included total fat mass (kg) and total body fat percentage (BF%), which was calculated as total fat mass divided by total DXA mass (fat and fat-free mass) and multiplied by 100.

### 2.3. Definitions

Age was calculated using date of birth and date of evaluation. Race was self-reported as either AA or Caucasian. BMI (weight [kg]/height [m]^2^) adjusted by age and sex was used to classify normal weight (<85th BMI%), overweight (≥85th and <95th BMI%), obesity (≥95th and <99th BMI%), and severe obesity (≥99th BMI%) [15]. The relative BMI formula was modified from the original method proposed by the Society of Actuaries [16,17] and West [18] since this concept was thought to allow the continuous comparison of severity of adiposity between sexes and age groups. RBMI was calculated as the actual BMI divided by the average BMI of the population (50th BMI% value) and multiplied by 100 [19]. RBMI cut points within the study sample were determined according to ROC curve analysis, with individuals classified as normal weight (RBMI < 100%), overweight (RBMI ≥ 100% and <120%), obesity (RBMI ≥ 120% and <160%), or severe obesity (RBMI ≥ 160%). BF% by DXA was used to compare BMI% and RBMI performance at different adiposity cut points. Though there is no standard for the normal BF% of adolescents, it is reasonable to apply the cut points proposed by Williams et al. (≥25% in males and ≥30% in females) [9].

### 2.4. Statistical Analysis

Analyses were performed using SPSS software v23.0 (IBM Corp., Armonk, NY, USA). The Kolmogorov–Smirnov test was conducted to assess the normality of continuous variables. Non-normally distributed variables were presented as median (interquartile range) and differences were evaluated using the Mann–Whitney U test. Proportions were presented as percentages with a 95% confidence interval (95% CI). Correlations of BF% with BMI% and with RBMI in the total sample, by sex and by race were performed using Spearman correlation and visualized using scatter plots. Measures of asymmetry were used to characterize the differences in data dispersion between BMI% and RBMI.

Receiver operating characteristics (ROC) curves, area under the curve (AUC), sensitivity, specificity, and Youden index were all calculated to compare the diagnostic performance of the BMI% and RBMI in detecting high body fat percentage (≥25 BF% in boys and ≥30 BF% in girls by DXA). As a sensitivity analysis, BMI% and RBMI performance were compared using >85th and >95th percentile BF% in both sexes. The highest sum sensitivity plus specificity favoring sensitivity (each value was >50%) was used to determine the optimal cutoff value of RBMI in detecting excess. The level of statistical significance was *p*-value < 0.05.

### 2.5. Ethical Considerations

All procedures were performed in accordance with the Helsinki Declaration. Prior to data collection in the original studies, youth assented to participate in the study and informed consent was obtained from each parent/legal guardian or from the youth, as applicable. This study, which used de-identified data from the previously conducted studies (IRB #7917 and #8365) was considered Non-Human Subject Research, and thus did not require Institutional Review Board approval.

## 3. Results

### 3.1. Baseline Characteristics

In total, 567 adolescents (74% female) were included in the analysis. The median age was 13.2 years (interquartile range: 12.0–14.7) with males being marginally older than females (*p* = 0.05). Males were also heavier (+31.2 kg) and taller (+5.8 cm) than female subjects, with an 11-point higher BMI, 50 percentage-point higher RBMI, and 6.7% more body fat. African Americans (AA, 37.0%) were older (14.7 vs. 12.5 year), heavier (94.5 vs. 46.4 kg), 7.7 cm taller, and had 16.2 kg/m^2^ more of BMI, with an RBMI 75.7 percentage points higher and 15.2 greater BF%, than Caucasians (*p* = 0.001 for all) (Table 1).

### 3.2. Correlation of BF% with BMI% and RBMI in Total Sample and by Sex and Race

**Figure 1:**
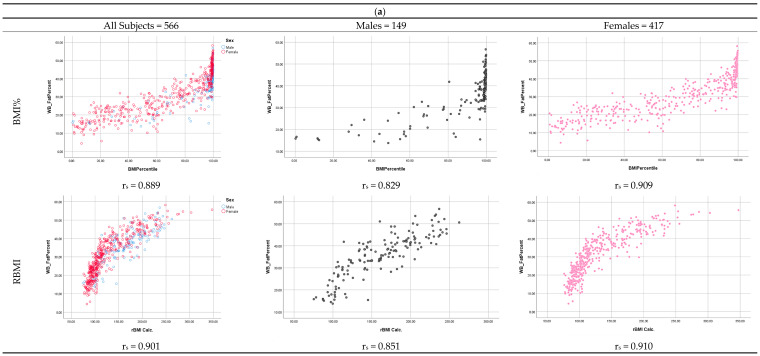

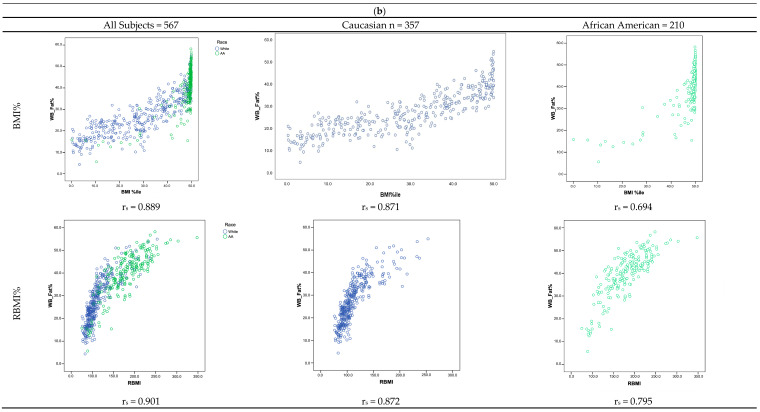
(**a**) Correlation between body fat percentage (BF%) and BMI% and RBMI in both sexes; Spearman correlation analysis was performed; (**b**) Correlation between body fat percentage (BF%) and BMI% and RBMI in both races; Spearman correlation analysis was performed. Abbreviations: BMI—Body mass index, RBMI—Relative body mass index, r_s_—Spearman’s rank correlation coefficient.

Adjusted by age, BF% showed strong positive correlations with both BMI% (r_s_ = 0.889) and RBMI (r_s_ = 0.901) for the total sample, as well as in male (BMI%, r_s_ = 0.829, RBMI, r_s_ = 0.851) and female subjects (BMI%, r_s_ = 0.909; RBMI, r_s_ = 0.910), respectively (Figure 1a). When stratified by race, BF% maintained its positive correlation with both BMI% and RBMI, in the Caucasian subsample (r_s_ = 0.871 and r_s_ = 0.872, respectively). In AA, the correlation coefficient was smaller between BF% and both BMI% and RBMI (r_s_ = 0.694 and r_s_ = 0.795, respectively; Figure 1b).

Independently of sex and race, trends in the relationship with BF% were different for both indices. BMI% exhibited a linear correlation with BF%, while the relationship between RBMI and BF% exhibited a curvilinear correlation pattern. However, it is worth noting that BMI percentiles represent a category and lack individual precision. BMI% ≥ 85th included subjects with a BF% range of more than 30, hindering correlations between BMI% and BF% within that category. RBMI exhibited lower levels of dispersion and better delineated the relationship with BF% independently of sex and race (Figure 1a,b). In comparing BMI% and RBMI data dispersion using quantified asymmetry, negative values represented a left-skewed distribution with increased dispersion, and a value near zero indicated reduced dispersion with values clustered around the median. For the entire sample, BMI% showed significant negative asymmetry compared to RBMI. The level of dispersion was even larger in those with a greater level of adiposity. BMI% showed two times greater dispersion in males than females, and nearly four times greater dispersion in AA as compared to Caucasians. All variables demonstrated a non-normal distribution with the exception of RBMI in AA (Table 2).

### 3.3. Comparing Performance of BMI to RBMI at Both Different Levels of BF% and Specific Cut Points to Detect and Categorize Increased BF% by Sex and Race

**Figure 2:**
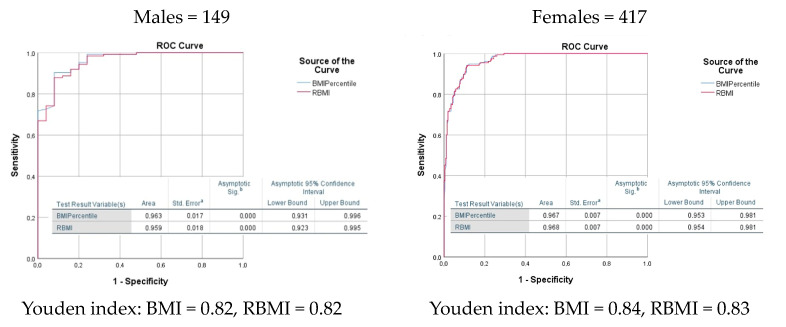
Receiver operating characteristic (ROC) curves for identifying high DXA total body fat percentage (BF%) by sex using BMI% and RBMI. Superscript a notates the Standard Error as being under the non-parametric assumption. Superscript b describes the asymptotic significance under a null hypothesis assuming a true area of 0.5.

An ROC curve analysis comparing area under the curve (AUC) as well as Youden Index showed similar performance between BMI% and RBMI to detect ≥25 BF% in males and ≥30 BF% in females (Figure 2). AUC was above 0.96 for both BMI and RBMI in both sexes with a 95% CI. Sensitivity analyses conducted for various BF% thresholds (≥15%, ≥20%, ≥35%, ≥40%, and ≥45%) for both sexes revealed no significant differences between BMI% and RBMI. Similarly, we performed sensitivity and specificity analysis to detect optimal RBMI cut points and categorize differing degrees of increased BF% levels in male and female subjects (Table 3). In males, the 85th BMI percentile detected BF% ≥ 25 and the 97th BMI percentile detected BF% ≥ 35. In females, the 85th BMI percentile detected ≥ 35 BF% and the 98th percentile detected ≥ 45 BF%. For RBMI, the best cut point to detect overweight (RBMI ≥100%–<120%), obesity (RBMI ≥120%–<160%), and severe obesity (RBMI ≥ 160%) were proposed for both sexes (Table 4). Using these RBMI cut points and BMI% categories, we evaluated the difference in the prevalence and distribution of adiposity categories (Table 5). In the total sample, RBMI identified 19.8% more adolescents as being overweight (OW) than BMI% (31.8% vs. 12.0%). Similarly, RBMI detected 14.2%, 21.3%, and 23.5% more adolescents as OW in males, females, and Caucasians, respectively. For the total sample, RBMI diagnosed 5.5% more subjects as having obesity (OB), which significantly increased among female (+7.4%) and Caucasian youth (+8.4%). The diagnosis of severe obesity (SVOB) increased by 4.2% when using RBMI in the total sample, although the change was only statistically significant among AA (+17.2%).

## 4. Discussion

Among relatively obese children, BMI% is a good indicator of excess adiposity, but its accuracy diminishes in relatively thin children [7,10,20,21,22,23]. In this study, we found that while both BMI% and RBMI are positively correlated to BF% after adjusting by age, their relationship pattern is dissimilar; a linear correlation with greater dispersion vs. the curvilinear relationship pattern of the RBMI with a lower level of dispersion (Figure 1a). The adjustment of an individual’s BMI by the 50th percentile value specific to their age and sex more closely fits the relationship between RBMI and BF%, allowing better assessment of BF%, especially in subjects with overweight and lower levels of adiposity (Figure 1). In this study, a BMI percentile ≥ 85th included subjects with widely discrepant BF% values, demonstrating the significant variability among those similarly classified by BMI%. Overall, the findings of this study support the use of RBMI as an alternative measure to BMI% for assessing adiposity in adolescents. The RBMI of a subject has an individual value representing the percentage of their current BMI relative to the 50th percentile value of the population, and also allows the comparison of the longitudinal changes of the same individual in short periods of time. For example, using BMI%, a girl with a BMI of 20 kg/m^2^ at ages 6, 7, 8, and 10 year will consistently be categorized as obese without reference to the progression of obesity until she reaches 10 year. RBMI transforms this measurement into a continuous variable reflecting the percentage of a patient’s excess BMI and the desired reference value. In this instance, the girl’s RBMI would be 131%, 128%, 126%, and 118% at ages 6, 7, 8, and 10 year, respectively, thereby objectively measuring excess adiposity and comparing its severity and evolution across ages.

Our results also suggested that the relationships between BMI% and RBMI with BF% were not significantly affected by sex, though females exhibited 6.7% less BF% (*p* = 0.001) and 37.8% less weight (*p* = 0.001) than males (Table 1). It is possible that this discrepancy may not be clinically relevant, as the weight difference between males and females was only 3.05 kg after being adjusted by age. The fact that both indices are adjusted by sex and age may also play a role in ameliorating disparities. However, we found that higher levels of adiposity are required to delineate the relationship between BF% and BMI%. When stratified by race, discrepancies in the severity of adiposity became more evident, as the Caucasian group was clearly leaner than AA, exhibiting 15.2% less BF% (*p* = 0.001) and 50.8% lower weight (*p* = 0.001) (Table 1). Among the total sample and within Caucasians, BF% exhibited a lowered correlation coefficient with BMI% (r = 0.693 vs. r = 0.807 and r = 0.614 vs. r = 0.807, respectively), while RBMI remained relatively unchanged even among leaner subjects (Figure 1b). As compared with Caucasians, AA had 14.17 kg more of fat, a measurement that is ~five times higher than the difference between sexes. Ethno-racial differences at equivalent levels of BMI-for-age have been previously described; Black children had less body fatness (mean, 3%) than white children [24]. However, the magnitude of such a discrepancy is very much lower than what we found in our sample, even after controlling by age. It has also been suggested that ethnicity did not significantly influence the BF%–BMI relation after first controlling for age and sex. BMI is age and sex dependent when used as an indicator of body fatness, but it is ethnicity independent in Black and White adults [8,25]. Our results are supported by previous studies and offer a better explanation for the larger discrepancy in the level of adiposity between Caucasian and AA adolescents. In our sample, almost 70% of AA subjects were classified as having SVOB by RBMI. In a study using DXA data from NHANES 1999–2006 (n = 10,465, aged 8–20 years old), Ryder et al. also demonstrated that BMI% was most accurate in detecting excess adiposity among adolescents with class 2 and 3 obesity, while significant discordance was observed among those with overweight or class 1 obesity [8]. Other similar analyses have confirmed that the degree of fatness modifies the relationship between BMI% and body fat mass [5,26,27].

Our study demonstrated that both BMI% and RBMI showed similar performance in detecting excessive BF% using cut points of ≥25 BF% in males and ≥30 BF% in females. In addition, sensitivity analyses using different levels of BF% did not show significant differences between the two indices (Figure 2). This study identified clinically useful RBMI cut points to categorize the severity and progression of obesity with higher sensitivity and specificity to identify different levels of BF% in youth of both sexes (Table 4). These findings favor the use of RBMI over BMI% in preventing misclassification of youth (Table 5), with close to 20% more youth who were classified as having normal weight by BMI% exhibiting excessive adiposity in the overweight range (males: BF% ≥ 20 to <25; females: BF% ≥ 25 to <35). The prevalence of youth meeting the BF% criteria for OB (males: BF% ≥ 25 to <35; females: BF% ≥ 35 to <45) also increased (+5.5%). Nevertheless, this only reached statistical significance among females and Caucasians, the groups with higher proportions misclassified as NW and OW by BMI% (+7.4% and +8.4%, respectively). Similarly, a higher proportion of subjects (+4.2%) met the BF% criteria for having SVOB (males: BF% ≥ 35; females: BF% ≥ 45), most of whom were AA (+17.2%).

The hallmark of obesity is excess adipose tissue [25]. BMI% is used as a non-invasive proxy measure for defining the level of adiposity [28]. Nonetheless, the accuracy of BMI% varies according to the degree of body fatness. A systematic review and meta-analysis of obesity in adolescents demonstrated that BMI has a sensitivity of only 73% to identify pediatric obesity, meaning that more than 25% of children with obesity would not be identified as having obesity [10]. Using RBMI, we were able to identify 29.5% more youth as having overweight or obesity than their BMI%-assigned categorization (+19.8% more OW, and +9.7% either OB or SVOB) (Table 5). We have previously reported that the ≥85th percentile threshold currently recommended for initial evaluation in clinical practice does not highlight the early health impact of adiposity in adolescents [13,19,29]. By the time adolescents are classified as having OW, the prevalence of cardiometabolic risk factors is significantly higher [30,31,32]. This misclassification will translate to irreversible delays to patient/parent awareness, as well as the loss of critical opportunities to mitigate the progression of the health risk associated with higher levels of adiposity. Another meta-analysis of 24 cohort studies demonstrated that skin fold testing or waist circumference did not perform better than BMI% for childhood obesity [33]. Thus, the implementation of RBMI in the pragmatic clinical setting will offset the limitation of BMI% as an indicator of excessive adiposity. It is important to have adequate tools in clinical practice to identify early stages of risk.

The limitations of this study are primarily associated with the distribution of the sample, including the lack of balance for age, weight, or BF%. In our sample, AA were significantly older and heavier, and demonstrated the highest BF%. Although males in this sample were also older and heavier than females, the differences between sexes were less evident than between racial groups. The results of the study cannot be adequately extrapolated to the general population, as it only included African American and Caucasian adolescents. Furthermore, the prevalence of subjects having NW was overrepresented among Caucasians and females, while SVOB mainly affected African Americans. The strengths of this study are the inclusion of AA and Caucasian adolescents with a wide range of adiposity, and the use of a methodology employed by several landmark studies to allow a better comparison of measures of adiposity. Another strength is the examination of clinically relevant adiposity measures (BMI% and RBMI) using DXA as the standardized method of BF% measurement.

## 5. Conclusions

Excess adipose tissue is the single best predictor both of adult obesity and of earlier development of associated complications. The BMI percentile measure underestimates the level of adiposity, especially at lower BF% values, resulting in misclassification and precluding clinicians from earlier intervention in at-risk populations. RBMI is an alternative measure for assessing adiposity in adolescents which improves the earlier detection of excess of adiposity in individuals who would otherwise be classified as having normal weight or overweight by BMI%. RBMI allows a more meaningful comparison of the longitudinal changes of adiposity over any period. This study lays the groundwork to better define and compare the relationship between different levels of BF% in the pragmatic clinical setting, and to evaluate its health impact on youth. Future studies using similar methodologies should aim to evaluate a larger sample that is more representative in terms of age, sex, and race, and thus can better inform conclusions about adiposity and its associations.

## Figures and Tables

**Table 1:** Characteristics of the studied population.

	Total	Males	Females	*p*	African Americans	Caucasians	*p*
N (%)	567 (100)	149 (26.3)	418 (73.7)		210 (37.0)	357 (63.0)	
Age (y)	13.2 (12.0–14.7)	14.0 (12.2–15.6)	12.9 (11.9–14.3)	0.05	14.7 (13.0–16.1)	12.5 (11.7–13.7)	<0.001
Weight (kg)	56.1 (43.1–90.4)	82.5 (54.2–112.2)	51.3 (41.1–76.0)	<0.001	94.5 (75.8–113.9)	46.4 (39.6–56.4)	<0.001
Height (cm)	157.8 (151.3–164.8)	162.8 (155.0–170.2)	157.0 (150.3–163.0)	<0.001	162.5 (157.5–169.4)	154.8 (149.2–161.9)	<0.001
BMI	22.4 (18.2–33.4)	31.3 (22.7–39.0)	20.4 (17.6–29.7)	<0.001	35.6 (29.3–41.3)	19.4 (17.2–22.5)	<0.001
BMI%	85.0 (48.7–99.7)	98.7 (86.5–99.5)	74.3 (36.5–97.1)	<0.001	99.9 (96.7–99.5)	60.5 (32.2–86.8)	<0.001
RBMI	120.0 (99.7–173.1)	161.6 (119.5–197.4)	111.4 (95.3–154.0)	<0.001	179.2 (150.6–209.9)	103.5 (98.6–120.6)	<0.001
BF% (DXA)	32.2 (22.8–41.0)	36.7 (29.3–42.1)	30.0 (21.6–39.6)	<0.001	41.4 (34.9–46.5)	26.2 (20.1–34.3)	<0.001

Continuous variables are median (interquartile range). Mann–Whitney test was used for difference in medians. BMI—Body Mass Index, BMI%—Body mass index percentile, RBMI—Relative Body Mass Index, BF%—Body Fat percentage, DXA—dual energy X-ray absorptiometry.

**Table 2:** Measures of central tendency, degree of dispersion, and distribution of BMI percentage (BMI%) and relative body mass index (RBMI) data in African American and Caucasian adolescents.

Measure	All Subjects		Males		Females		African Americans		Caucasians	
	BMI%	RBMI	BMI%	RBMI	BMI%	RBMI	BMI%	RBMI	BMI%	RBMI
Mean (95% CI)	71.3 (68.8–73.9)	137.4 (133.4–141.4)	87.4 (83.7–91.0)	159.8 (152.2–167.3)	65.6 (62.5–68.6)	129.4 (124.9–133.8)	93.1 (91.8–96.0)	179.8 (173.7–185.8)	58.1 (54.9–61.2)	112.4 (109.3–115.4)
Median	85.0	118.7	98.7	161.4	74.3	11.1	99.0	179.2	60.5	103.5
Standard Deviation	31.24	48.3	22.5	46.7	31.9	46.4	15.7	44.3	30.5	29.3
Min–Max	0.2–99.9	75.3–347.9	0.2–99.9	75.3–252.3	0.7–99.9	76–347	0.2–99.9	75.3–272.6	0.7–99.7	76–253
Asymmetry	−0.75	0.95	−2.2	0.11	−0.52	1.38	−3.93	0.155	−0.25	1.96
Kolgomorov Test	0.0001 *	0.0001 *	0.0001 *	0.02 *	0.0001 *	0.0001 *	0.0001 *	0.20	0.0001 *	0.0001 *

* Does not have a normal distribution. CI: Confidence Intervals. BMI%: Body mass index percentile. RBMI: Relative body mass index.

**Table 3:** Sensitivity and specificity analysis to select the best of relative body mass index (RBMI) cut point in identifying different levels of DXA body fat percentage (BF%) in both sexes.

Cut point ≥ 25% total body fat	Total				Males				Females			
	RBMI	Sen	Spe	Sum	RBMI	Sen	Spe	Sum	RBMI	Sen	Spe	Sum
	**110.11**	**84.47**	**95.16**	**179.63**	114.76	89.52	84.00	173.52	105.67	87.50	91.93	179.43
	110.43	84.21	95.16	179.37	115.19	88.71	84.00	172.71	105.72	87.11	91.93	179.03
	110.87	83.95	95.16	179.11	116.35	88.71	88.00	176.71	105.76	87.11	92.55	179.66
	111.05	83.68	95.16	178.85	117.17	87.90	88.00	175.90	105.90	87.11	93.17	180.28
	111.10	83.42	95.16	178.58	**117.54**	**87.90**	**92.00**	**179.90**	**106.05**	**87.11**	**93.79**	**180.90**
	111.21	83.16	95.16	178.32	118.38	87.10	92.00	179.10	106.12	86.72	93.79	180.51
	111.37	82.89	95.16	178.06	119.25	86.29	92.00	178.29	106.58	86.33	93.79	180.12
	111.48	82.63	95.16	177.79	123.01	85.48	92.00	177.48	107.09	85.94	93.79	179.73
	111.62	82.37	95.16	177.53	126.98	84.68	92.00	176.68	107.26	85.55	93.79	179.34
	111.74	82.11	95.16	177.27	127.78	83.87	92.00	175.87	107.40	85.55	94.41	179.96
Cut point ≥ 30% total body fat	Total				Males				Females			
	RBMI	Sen	Spe	Sum	RBMI	Sen	Spe	Sum	RBMI	Sen	Spe	Sum
	109.51	94.97	85.08	180.05	117.54	93.52	75.61	169.13	109.21	94.29	88.89	183.17
	109.54	94.65	85.08	179.73	118.38	92.59	75.61	168.20	109.46	94.29	89.37	183.66
	109.59	94.65	85.48	180.14	119.25	92.59	78.05	170.64	109.54	93.81	89.37	183.18
	109.66	94.65	85.89	180.54	123.01	91.67	78.05	169.72	109.59	93.81	89.86	183.66
	**109.80**	**94.65**	**86.29**	**180.94**	**126.98**	**91.67**	**80.49**	**172.15**	**109.75**	**93.81**	**90.34**	**184.15**
	109.89	94.34	86.29	180.63	127.78	90.74	80.49	171.23	109.89	93.33	90.34	183.67
	109.93	94.03	86.29	180.32	129.33	89.81	80.49	170.30	109.93	92.86	90.34	183.20
	110.00	94.03	86.69	180.72	130.85	88.89	80.49	169.38	110.00	92.86	90.82	183.68
	110.07	93.71	86.69	180.40	131.49	87.96	80.49	168.45	110.07	92.38	90.82	183.20
	110.11	93.40	86.69	180.09	132.48	87.04	80.49	167.52	110.11	91.90	90.82	182.73
Cut point ≥ 85th percentile total body fat	Total				Males				Females			
	RBMI	Sen	Spe	Sum	RBMI	Sen	Spe	Sum	RBMI	Sen	Spe	Sum
	171.93	87.50	91.97	179.47	147.94	85.23	83.61	168.83	134.23	92.38	87.50	179.88
	172.76	87.50	92.24	179.74	148.57	84.09	83.61	167.70	134.55	92.38	87.82	180.20
	173.61	87.50	92.52	180.02	149.60	82.95	83.61	166.56	134.91	92.38	88.14	180.52
	175.32	87.50	92.80	180.30	150.09	82.95	85.25	168.20	135.04	92.38	88.46	180.84
	**176.86**	**87.50**	**93.07**	**180.57**	**152.48**	**82.95**	**86.89**	**169.84**	**135.74**	**92.38**	**88.78**	**181.16**
	177.21	85.71	93.07	178.79	156.98	81.82	86.89	168.70	136.48	91.43	88.78	180.21
	177.47	83.93	93.07	177.00	159.30	80.68	86.89	167.57	136.58	90.48	88.78	179.26
	177.91	83.93	93.35	177.28	159.90	79.55	86.89	166.43	136.61	89.52	88.78	178.31
	178.69	83.93	93.63	177.56	160.78	78.41	86.89	165.29	136.84	89.52	89.10	178.63
	179.21	82.14	93.63	175.77	161.38	77.27	86.89	164.16	137.15	89.52	89.42	178.95
Cut point ≥ 95th percentile total body fat	Total				Males				Females			
	RBMI	Sen	Spe	Sum	RBMI	Sen	Spe	Sum	RBMI	Sen	Spe	Sum
	150.34	95.12	83.52	178.64	170.52	91.07	82.80	173.87	150.02	92.54	86.57	179.11
	150.57	95.12	83.75	178.87	171.31	91.07	83.87	174.94	150.57	92.54	86.86	179.39
	151.73	95.12	83.97	179.09	171.92	91.07	84.95	176.02	151.73	92.54	87.14	179.68
	152.95	95.12	84.20	179.32	172.74	91.07	86.02	177.09	152.95	92.54	87.43	179.97
	**153.07**	**95.12**	**84.42**	**179.55**	**173.90**	**91.07**	**87.10**	**178.17**	**153.07**	**92.54**	**87.71**	**180.25**
	153.30	94.31	84.42	178.73	174.63	89.29	87.10	176.38	153.30	91.04	87.71	178.76
	154.16	94.31	84.65	178.96	174.76	89.29	88.17	177.46	154.43	91.04	88.00	179.04
	155.10	93.50	84.65	178.15	175.00	87.50	88.17	175.67	155.64	91.04	88.29	179.33
	155.64	93.50	84.88	178.37	175.19	85.71	88.17	173.89	156.61	91.04	88.57	179.62
	156.61	93.50	85.10	178.60	175.98	83.93	88.17	172.10	157.40	89.55	88.57	178.12

Boxed, bolded rows represent selected cut point values. Abbreviations: RBMI—Relative body mass index percentile, Sen—Sensitivity, Spe—Specificity, Sum—Sum of Sensitivity and Specificity.

**Table 4:** Proposed relative body mass index (RBMI) cut points to identify high adiposity in adolescents.

Categories		Males			Females			Both Sexes
Traditional	Adiposity by BF%	BF Cut Points (%)	Reported RBMI Cut Points (%)	Proposed RBMI Cut Points (%)	BF Cut Points (%)	Reported RBMI Cut Points (%)	Proposed RBMI Cut Points (%)	Proposed RBMI Cut Points (%)
NW	Normal Adiposity	<20	<100	≥75–<100	<25	<106.1	≥75–<110	≥75–<100
OW	Mildly high Adiposity	≥20–<25	<117.6	≥100–<120	≥25–<35	<117.7	≥110–<120	≥100–<120
OB	Moderately high adiposity	≥25–<35	<161.7	≥120–<160	≥35–<45	<160.0	≥120–<160	≥120–<160
SVOB	Severely high adiposity	≥35	≥161.7	≥160	≥45	≥160.0	≥160	≥160

BMI%—Body mass index percentile, RBMI—Relative body mass index, NW—Normal weight, OW—Overweight, OB—Obesity, SVOB—Severe obesity, BF%—Body fat percentage by DXA.

**Table 5:** Comparing prevalence of adiposity categories using BMI% and RBMI selected cut points.

	Adiposity Categories	Total (n = 567)	Males (n = 149)	Females (n = 418)	African Americans (n = 210)	Caucasians (n = 357)
BMI%	NW (<85th %ile)	65.9 (62.0–69.6)	40.2 (32.0–48.8)	72.8 (68.6–76.6)	9.5 (6.2–14.2)	73.7 (68.9–78.0)
	OW (≥85th and <95th %ile)	12.0 (9.6–14.8)	11.8 (7.3–18.6)	12.0 (9.4–15.3)	8.1 (5.1–12.6)	12.4 (9.1–15.8)
	OB (≥95th BMI–<99th %ile)	11.5 (9.2–14.3)	24.4 (17.8–32.6)	8.0 (5.9–10.8)	31.0 (25.1–37.5)	9.2 (6.7–12.7)
	SVOB (≥99th %ile)	10.6 (8.4–13.4)	23.6 (17.1–31.7)	7.2 (5.2–9.9)	51.4 (44.7–58.1)	5.0 (3.2–7.8)
RBMI	NW (<100%)	36.4 (32.7–40.4)	21.3 (15.0–29.2)	40.5 (36.2–45.0)	3.8 (1.9–7.3)	37.8 (32.9–42.9)
	OW (≥100 <120%)	31.8 (28.2–35.6)	26.0 (19.1–34.2)	33.3 (29.2–37.7)	6.2 (3.6–10.3)	35.9 (31.0–40.9
	OB (≥120 <160%)	17.0 (14.2–20.2)	22.8 (16.4–30.8)	15.4 (12.4–18.9)	21.4 (16.4–27.5)	17.6 (14.0–21.9)
	SVOB (≥160%)	14.8 (12.2–17.8)	29.9 (22.6–38.4)	10.8 (8.3–13.8)	68.6 (61.5–74.0)	8.7 (6.2–12.1)

Frequencies are expressed as percentages and 95% confidence intervals (95% CI), and differences were considered when no 95% CI overlap was detected. BMI%—Body Mass Index Percentile, RBMI—Relative Body Mass Index, NW—Normal weight, OW—Overweight, OB—Obesity, SVOB—Severe obesity, %ile—Percentile.

## Data Availability

The data that support the findings of this study are available from the corresponding author (PVM) upon reasonable request.
